# Application of the steady-state intestinal perfusion system in measuring intestinal fluid absorption and bicarbonate secretion *in vivo*

**DOI:** 10.3389/fphys.2023.1163888

**Published:** 2023-07-11

**Authors:** Wenjuan Fan, Qinghai Tan

**Affiliations:** Department of Gastroenterology, Tongji Hospital, Tongji Medical College, Huazhong University of Science and Technology, Wuhan, Hubei, China

**Keywords:** intestinal fluid absorption, intestinal bicarbonate secretion, steady-state intestinal perfusion system, *in vivo*, HCO_3_^-^

## Abstract

**Background:** The steady-state intestinal perfusion system represents a tool used in measuring intestinal fluid absorption and bicarbonate secretion *in vivo*; however, detailed procedures and parameters were not elucidated fully.

**Aim:** We focused on the methods of the steady-state intestinal perfusion system comprehensively including the blood pressure, hematocrit, blood gas, and heart rate of mouse.

**Methods:** Anesthetized, tracheally intubated, and artificially ventilated mice were used for this system. The blood pressure, hematocrit, blood gas, heart rate, and rate of fluid absorption and HCO_3_^-^ secretion of the small intestine and colon at different time points were evaluated.

**Results:** Blood pressure, hematocrit, blood gas, and heart rate became stable at the 30 min time point after completion of surgery and could be maintained for 2 h. Rates of fluid absorption and bicarbonate secretion were also kept stable during the period of steady state of mice. Rates of fluid absorption and bicarbonate secretion were different among the jejunum, ileum, proximal, and mid-distal colon.

**Conclusion:** The steady-state intestinal perfusion system is a reliable system for measuring intestinal fluid absorption and bicarbonate secretion *in vivo.*

## Highlights

### New and noteworthy

1. The steady-state intestinal perfusion system is reliable in measuring intestinal fluid absorption and bicarbonate secretion *in vivo*
2. Blood pressure, hematocrit, heart rate, and blood gas are good parameters for monitoring the status of mice during the experiment
3. Rates of fluid absorption and bicarbonate secretion are different in different intestinal segments and could be affected by the pH of perfusates

## Introduction

The intestinal micro-environment in homeostasis is critical for maintaining normal body function. Notably, fluid transport and bicarbonate (HCO_3_^-^) secretion across intestinal epithelia are the two main items implicated in this process (Kato and Romero, 2011). Breaking the balance of fluid transport and bicarbonate secretion will result in diarrhea (or constipation) and dysbiosis (Sayuk et al., 2022). To decipher the mechanisms of dysfunction regarding fluid transport and bicarbonate secretion, undoubtedly, a perfect animal model is necessary.

A variety of studies have demonstrated that many transporters and channels, such as sodium hydrogen exchanger 3 (NHE3), the cystic fibrosis transmembrane conductance regulator (CFTR), and downregulated in adenoma (DRA), act in concert to regulate the process of fluid secretion and acid–base secretion in the digestive tract (Seidler and Nikolovska, 2019). To understand the physiological and pathophysiological functions of these transporters and channels, many *in vivo* animal models, such as steady-state intestinal perfusion systems, closed-loop techniques, fistulous animal models, enteropooling models, and stool water content measurements, have been created (Mourad, 2004). Among them, the fistulous animal model is used widely in dogs and pigs. Enteropooling models (luminal fluid measurement in the small intestine from the pylorus to the ileocecal junction, or the entire small and large intestine) and stool water content measurement can only give us a gross idea about the intestinal fluid transport with a limitation to discern functional differences along the entire intestine. In addition, compensatory colonic hyper-absorption typically masks the small intestinal fluid loss unless the latter is massive. Moreover, different expression patterns of ion transporters and channels are found along the entire intestinal tract (Xia et al., 2014; Seidler and Nikolovska, 2019). Thus, it is important to study the individual function of these ion transporters by measuring fluid transport in the different intestinal segments. Although the closed-loop technique can measure the fluid absorption and HCO_3_^-^ secretion in different intestinal segments (Jung et al., 2014), unstable data would be compromised because of the non-repeatability in one mouse of every experiment. Thus, setting an effective animal model that can measure fluid transport and HCO_3_^-^ secretion in the different intestinal segments of individual mouse repeatedly is necessary. Fortunately, a steady-state intestinal perfusion system can represent the proper tool (Liu et al., 2020). Many studies have reported to apply this experimental tool to evaluate the rate of fluid absorption and HCO_3_^-^ secretion in each mouse; however, regarding the detailed procedures of it, most studies just gave a brief description.

In this study, we focused on the methods of the steady-state intestinal perfusion system comprehensively including the blood pressure, hematocrit, blood gas, and heart rate of each mouse. In addition, the rate of fluid absorption and HCO_3_^-^ secretion at different time points was also measured to give a comprehensive impression to other researchers of interest.

## Methods

### Materials

All reagents were purchased from Sigma-Aldrich (Deisenhofen, Germany) and AppliChem (Darmstadt, Germany) unless mentioned otherwise. Isoflurane (Forane) was from Abbott, Wiesbaden, Germany.

### Mice

Mice with c57 background were approved by the Committee of Ethics of the Hannover Medical School Committee and the Tongji Hospital Committee assembled by the local government. Experiments were performed with age-matched and sex-matched littermates at the age of 12 weeks.

### Setting of the steady-state intestinal perfusion system animal model

Fluid absorption and bicarbonate secretion experiments were performed as previously described: Mice were put in a closed box gassed with air containing 5% isoflurane for anesthesia induction. Tracheal intubation was conducted after the mice got deep anesthesia and connected to a mechanical ventilator (MiniVent Type 845, Hugo Sachs Elektronik, March Hugstetten, Germany). Then, anesthetized mice were placed on a heating pad to keep the body temperature at around 37.5°C, which was measured by using a rectal thermistor probe (except the mid-distal colon due to the anus was occupied by a catheter for outflow collection). Respiration rate and tidal volume (TV) were set based on the weight of the mouse according to the recommendations from the “Operating Instructions for the Mouse Ventilator MiniVent Type 845.” The respiration rate ranged from 140 to 150/min, and tidal volume (TV) ranged from 200 to 250 μl. The intensity of anesthesia was tested via pedal withdrawal reflex. The isoflurane concentration in the anesthetic gas was reduced from 5% to 2.5% after the pedal withdrawal reflex disappeared.

A catheter was placed in the left carotid artery to monitor BP and heart rate and to infuse an isotonic Na_2_CO_3_ solution with a concentration of 100 mM at the rate of 0.1 ml/(10 g h) to keep the systemic acid–base balance. Another catheter was placed in the left femoral vein for continuous infusion of saline solution at the rate of 0.2 ml/h to compensate for fluid loss during the process of experimentation for ensuring hematocrit stability throughout the experiment.

The abdomen was dissected along the abdominal midline. To prepare the jejunal segment, the intestine was closed at the distance of 8–10 cm from the pylorus for the prevention of the pancreatic juice and bile entering the intestinal segment. A small incision was made close to the ligature along the anti-mesentery side, and a small polyethylene tube (inner diameter 1 mm) was inserted and secured by a ligature to pump solution for influx. Another small incision was made along the anti-mesentery side 3 cm from the former incision, and a small polyethylene cannula (inner diameter 2 mm) was inserted and secured by a ligature for outflow collection. To prepare the ileal segment, a small incision was made close to the cecum along the anti-mesentery side and a small polyethylene cannula (inner diameter 2 mm) was inserted and secured by the ligature to collect outflow. Another small incision was made along the anti-mesentery side 3 cm from outflow incision, and a small polyethylene tube (inner diameter 1 mm) was inserted and secured by the ligature for influx.

**FIGURE 1:**
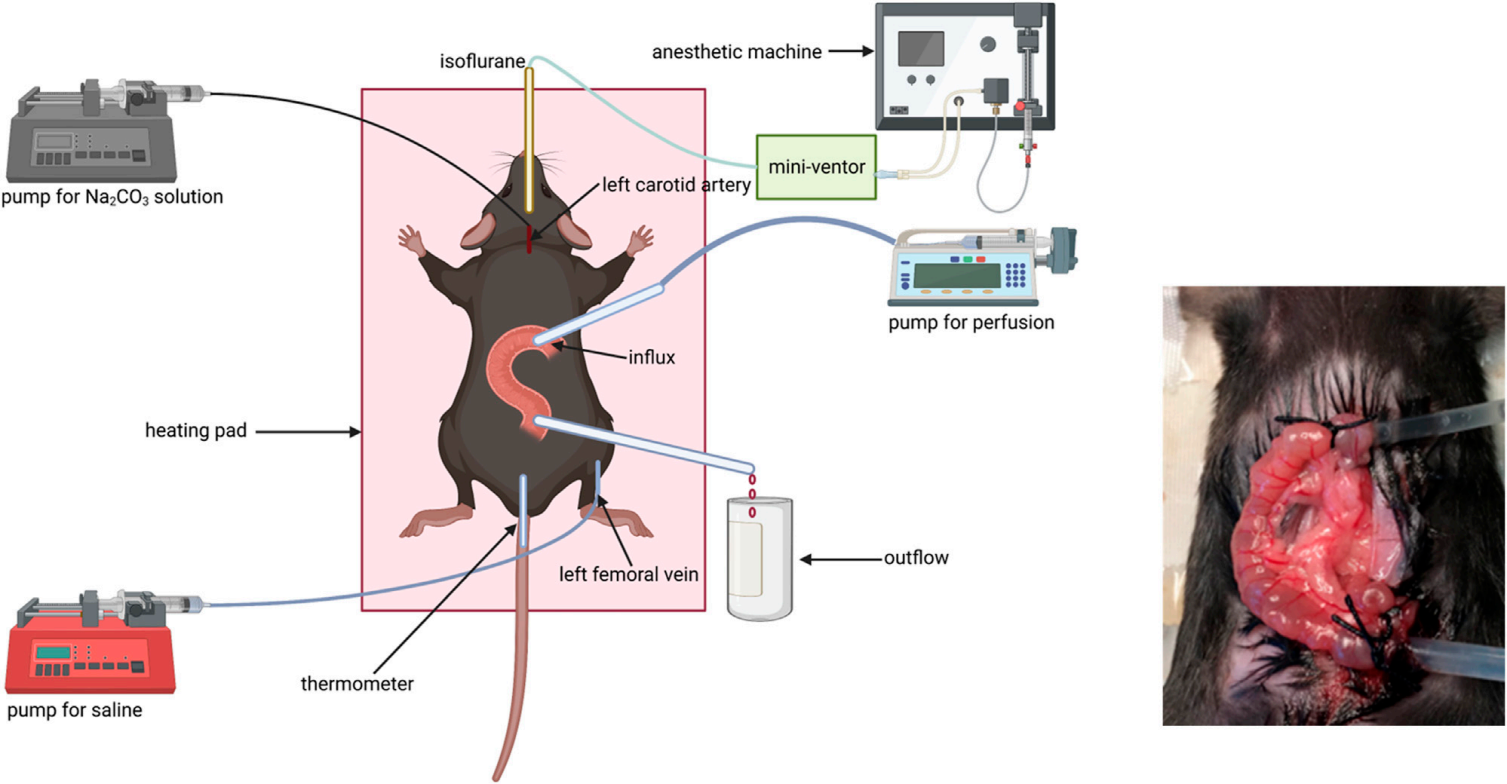
Schematic diagram of the animal model.

To prepare the proximal colonic segment, the influx incision was made close to the cecum and the outflow incision approximately 2 cm distally. To prepare the mid-distal colonic segment, the influx incision was made in the middle of the colon (∼4 cm from the cecum) and the outflow catheter was advanced through the anus and secured intraperitoneally. Each intestinal segment with intact blood supply was gently flushed before perfusion. Fluid absorption and bicarbonate secretion were measured after cardiovascular, respiratory, and intestinal functions got stabilized. The intestinal segment was perfused with an unbuffered solution with pH 7.4, 37°C, consisting of NaCl 145.5 mM, KCl 4.0 mM, and CaCl_2_ 1.2 mM, at a rate of 30 ml/h three times. All effluents from the isolated intestinal segment were visually free of bile and blood throughout all experiments (Figure 1).

### Measurement of heart rate and blood pressure

A catheter was placed in the left carotid artery and connected to a pressure sensor that could input the signal to LabChart 7 software. Blood pressure and heart rate were monitored by LabChart 7 automatically.

### Fluid absorption rate calculation

The rate of fluid absorption was calculated according to the following formula based on the weight of the influx and the outflow (effluent). R1 = (W1–W2). L^−1^. T^−1^. ρ^−1^, R1: the rate of fluid absorption (μl/cm.h), T: time (h), W1: the weight of influx (g), W2: the weight of outflow (g), L: the length of the segment (cm), and ρ: density of the fluid (ρ≈ 1 g. cm^−3^).

### Measurement of bicarbonate secretion

The determination of bicarbonate was conducted as described previously (Tan et al., 2021). The rate of luminal alkalization was calculated via the back titration of the effluents to pH 5.0 with 5-mM HCl in the following equation: R2 = (V2–V1). ρ/M^−1^. L^−1^. T^−1^, R2: the rate of bicarbonate secretion (uM/cm.h), V1: the volume of HCl used for influx, V2: the volume of HCl used for outflow, T: time, L: the length of the segment, M: relative molecular mass, and ρ: density of the solution.

### Blood gas and hematocrit analysis

Arterial blood samples were taken from the left carotid artery at different time points and used for blood gas analysis and hematocrit measurement. A 50 µl blood sample was collected in the capillary and transferred to a radiometer blood-gas analyzer (Radiometer, Copenhagen) for analysis automatically. Another 50 µl blood sample was also collected in the capillary that was sealed at one point and placed in a hematocrit centrifuge (Hematokrit 210, Hettich, Kirchlengern, Germany) at a rate of 1200 r/min for analysis, whose results were defined according to the scale on the panel of the hematocrit centrifuge machine.

### Statistics

Data are presented as means ± SEM, with the number of experiments given in parenthesis. Student’s t-test was used for quantitative data analysis. Two-sided *p* < 0.05 was considered statistically significant. Statistical analyses were conducted via SPSS software (version 21.0) and Prism 6.0 (GraphPad Software). For comparison of basal rates of fluid absorption and bicarbonate secretion between different intestinal segments within one genotype, the ANOVA for multiple comparisons was conducted (*p < 0.05).

## Results

### Vital signs at different time points of the experimental process

**FIGURE 2:**
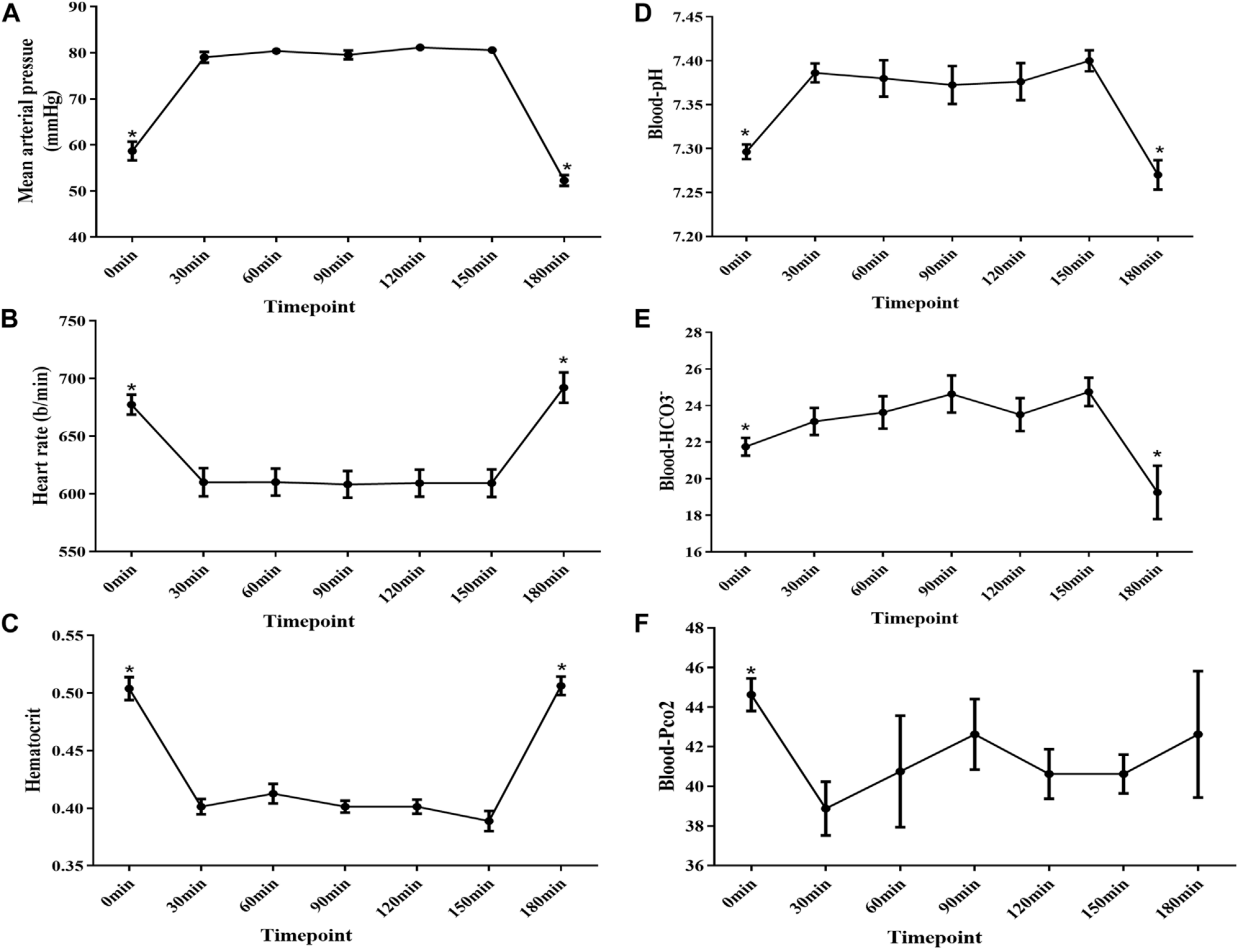
Mean arterial pressure , heart rate, hematocrit, and blood gas analysis at different time points after surgery completion. **(A)** MAP was below 60 mmHg at the 0 min time point, and the MAP increased to approximately 80 mmHg after 30 mins and was kept stable from 30 to 150 min time points. However, MAP decreased to 50 mmHg at the 180 min time point. **(B)** HR increased to approximately 677 b/min at the 0 min time point, and it decreased to approximately 610 b/min after 30 mins and was kept stable from 30 to 150 min time points. Last, it increased to 690 b/min at the 180 min time point. **(C)** Hematocrit increased to approximately 50% at the 0 min time point, and it decreased to approximately 41% after 30 mins and was kept stable from 30 to 150 min time points. Last, it increased to approximately 53% at the 180 min time point again. **(D–F)** Blood gas analysis showed that the mice got acidosis at the 0 min time point and recovered after 30 min and remained stable for 2 h. However, the mice got acidosis again at the 180 min time point (n = 8, NS: no significance, *p < 0.05).

Since the body temperature was maintained by the heating pad at around 37°C, and the tidal volume and breathing rate were controlled by using the mini-vent machine as seen in Methods. To figure out the optimized status for the next measurement of fluid absorption and HCO_3_^−^ secretion, we evaluated the blood pressure, heart rate, hematocrit, and blood gas at different time points after completion of surgery. The cardiovascular system was unstable with a high heart rate, low blood pressure, high hematocrit, and low arterial blood pH at the 0 min time point. Notably, after 30 min, the cardiovascular system became stable and could be maintained for 2 h. However, the status of mice deteriorated after 2 h (Figure 2).

### Rate of fluid absorption and bicarbonate secretion at different time points

**FIGURE 3:**
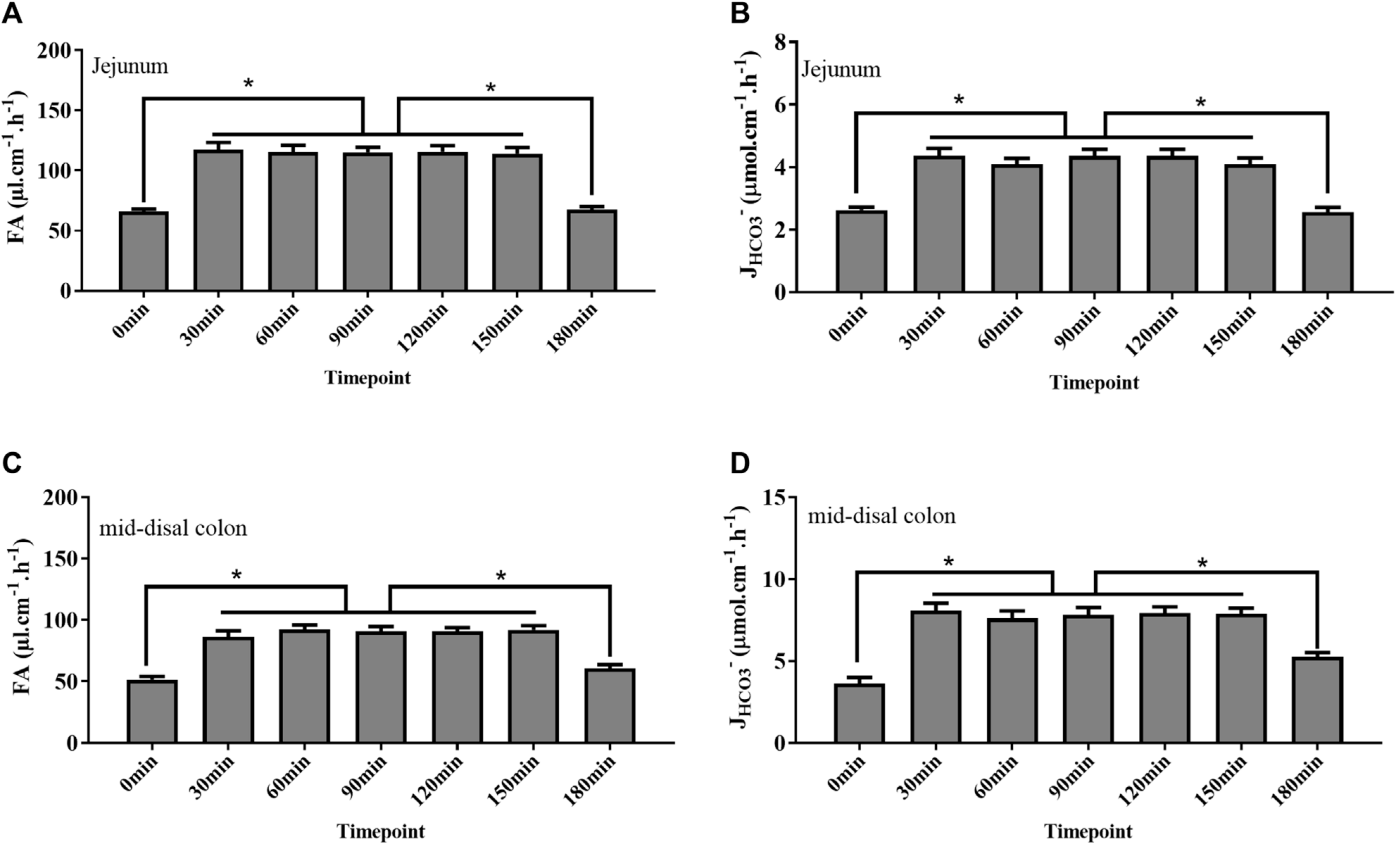
Rate of fluid absorption (FA) and bicarbonate secretion (*J*_*HCO3*_ *-*) at different time points after surgery completion. **(A, B)** Rate of FA and *J*_*HCO3*_ *-* in the jejunum at the 0 min time point was as low as 65 μl cm^−1^ h^−1^ and 2.6 μmol cm^−1^ h^−1^, respectively. After 30 mins, the rate of FA and *J*_*HCO3*_ *-* increased to 117 μl cm^−1^ h^−1^ and 4.3 μmol cm^−1^h^−1^, respectively, and kept stable from 30 to 150 min time points. At the 180 min time point, the rate of FA and *J*_*HCO3*_ *-* decreased to 67 μl cm^−1^h^−1^ and 2.5 μmol cm^−1^h^−1^, respectively. **(C, D)** Rate of FA and *J*_*HCO3*_ *-* in the colon at the 0 min time point was as low as 51 μl cm^−1^h^−1^ and 3.6 μmol cm^−1^h^−1^, respectively. After 30 mins, the rate of FA and *J*_*HCO3*_ *-* increased to 92 μl cm^−1^h^−1^ and 7.8  μmol cm^−1^h^−1^, respectively, and kept stable from 30 to 150 min time points. At the 180 min time point, the rate of FA and *J*_*HCO3*_ *-* decreased to 60 μl cm^−1^h^−1^ and 5.2 μmol cm^−1^h^−1^, respectively (n = 8, NS: no significance, *p < 0.05).

Studies have proved that holistic homeostasis of the body is critical for intestinal function, such as water movement and acid and base transport across the intestinal surface. To get stable data regarding fluid absorption and bicarbonate secretion, we tested their rate at different time points in the jejunum and mid-distal colon. The rate of fluid absorption and bicarbonate secretion was the lowest at 0, 180 min time point, corresponding to the low blood pressure at the same time point. However, the rate increased at 30 min and remained stable for approximately 2 h (Figure 3).

### Measurement of fluid absorption and bicarbonate secretion in different intestinal segments

**FIGURE 4:**
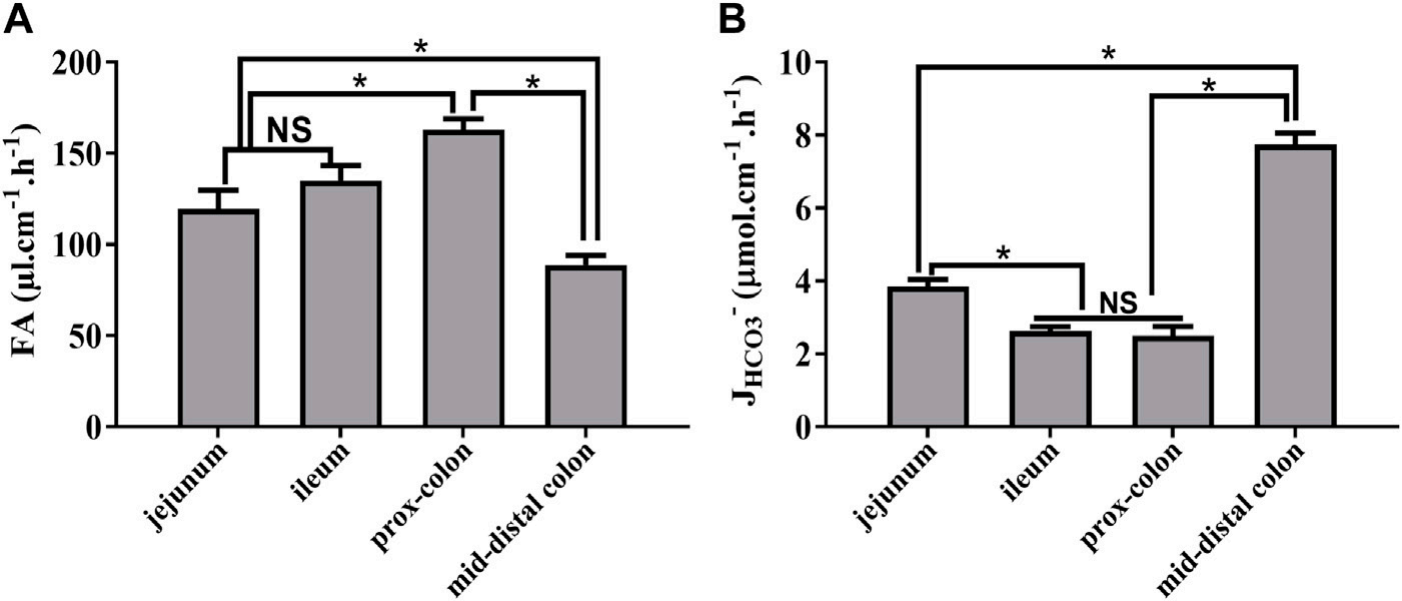
Rate of fluid absorption and bicarbonate secretion in different intestinal segments during the stable period. **(A)** Rate of FA in the jejunum, ileum, proximal colon, and mid-distal colon was 119.4 μl cm^−1^h^−1^, 135.1 μl cm^−1^h^−1^, 162.9 μl cm^−1^h^−1^, and 88.7 μl cm^−1^h^−1^, respectively. **(B)** Rate of *J*_*HCO3*_ *-* in the jejunum, ileum, proximal colon, and mid-distal colon was 3.8 μmol cm^−1^h^−1^, 2.6 μmol cm^−1^h^−1^, 2.4 μmol cm^−1^h^−1^, and 7.7 μmol cm^−1^h^−1^, respectively (n = 8, NS: no significance, *p < 0.05).

Next, we measured the fluid absorption and bicarbonate secretion in different intestinal segments during the stable time course (30–150 min). As shown in Figure 4, the rate of fluid absorption and *J*_*HCO3*_ *-* is different among the different segments with the highest rate of fluid absorption and *J*_*HCO3*_ *-* in the proximal colon and mid-distal colon, respectively (Figure 4).

### Effect of the pH of the perfusate on fluid absorption and bicarbonate secretion

**FIGURE 5:**
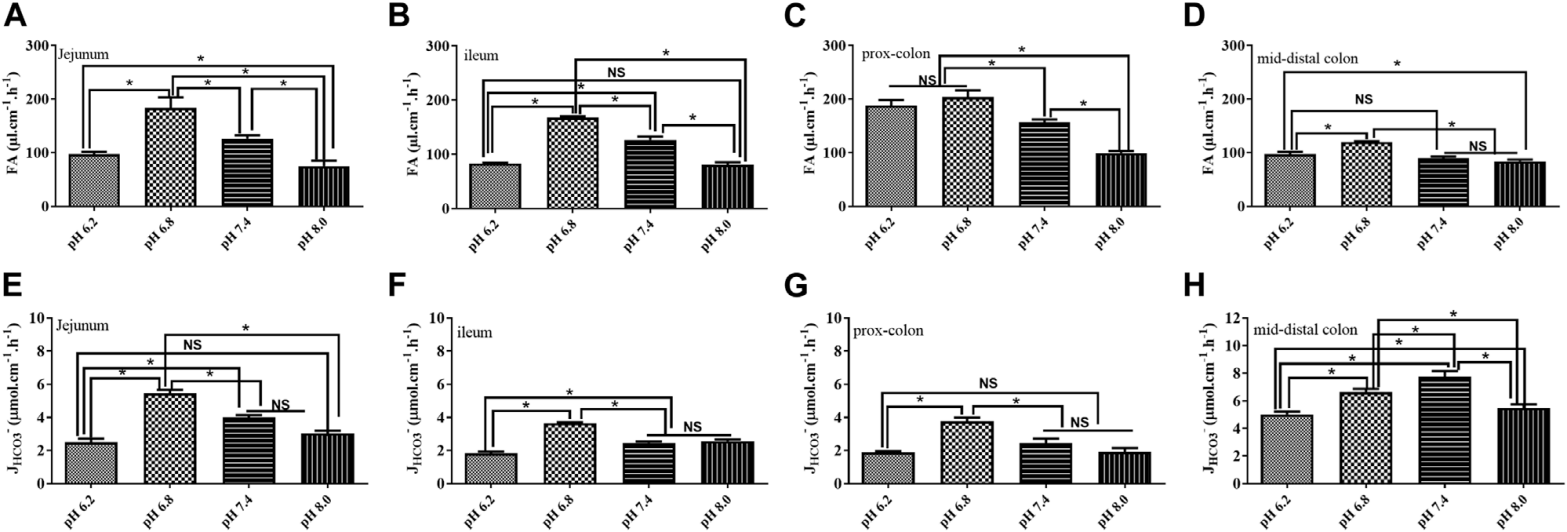
Effect of the pH of the perfusate on fluid absorption and bicarbonate secretion. **(A)** Rate of FA in the jejunum was 97.0 μl cm^−1^h^−1^, 183.4 μl cm^−1^h^−1^, 125.9 μl cm^−1^h^−1^, and 74.8 μl cm^−1^h^−1^ at the pH of 6.2, 6.8, 7.4, and 8.0, respectively. **(B)** Rate of FA in the ileum was 82.6 μl cm^−1^h^−1^, 167.5 μl cm^−1^h^−1^, 126.4 μl cm^−1^h^−1^, and 80.4 μl cm^−1^h^−1^ at the pH of 6.2, 6.8, 7.4, and 8.0, respectively. **(C)** Rate of FA in the proximal colon was 187.7 μl cm^−1^h^−1^, 203.4 μl cm^−1^h^−1^, 156.7 μl cm^−1^h^−1^, and 98.8 μl cm^−1^h^−1^ at the pH of 6.2, 6.8, 7.4, and 8.0, respectively. **(D)** Rate of FA in the proximal colon was 97.07 μl cm^−1^h^−1^, 119.6 μl cm^−1^h^−1^, 89.5 μl cm^−1^h^−1^, and 83.9 μl cm^−1^h^−1^ at the pH of 6.2, 6.8, 7.4, and 8.0, respectively. **(E)** Rate of *J*_*HCO3*_ *-* in the jejunum was 2.5 μmol cm^−1^h^−1^, 5.5 μmol cm^−1^h^−1^, 3.9 μmol cm^−1^h^−1^, and 3.04 μmol cm^−1^h^−1^. **(F)** Rate of *J*_*HCO3*_ *-* in the ileum was 1.8 μmol cm^−1^h^−1^, 3.6 μmol cm^−1^h^−1^, 2.4 μmol cm^−1^h^−1^, and 2.5 μmol cm^−1^h^−1^. **(G)** Rate of *J*_*HCO3*_ *-* in the proximal colon was 1.8 μmol cm^−1^h^−1^, 3.7 μmol cm^−1^h^−1^, 2.4 μmol cm^−1^h^−1^, and 1.9 μmol cm^−1^h^−1^. **(H)** Rate of *J*_*HCO3*_ *-* in the proximal colon was 5.0 μmol cm^−1^h^−1^, 6.6 μmol cm^−1^h^−1^, 7.7 μmol cm^−1^h^−1^, and 5.5 μmol cm^−1^h^−1^ (n = 5, NS: no significance, *p < 0.05).

To mimic the physiological status, usually, we used a solution with the pH at 7.4 for perfusion. However, a previous study demonstrated that the pH of the solution could affect the fluid absorption in the jejunum (Tan et al., 2021). In our study, we tested the effect of the un-buffered solution with different pH values on fluid absorption and bicarbonate secretion in different segments. As shown in Figure 5, the rate of fluid absorption increased in all segments’ perfusate with pH 6.8. Regarding *J*_*HCO3*_ *-*, the rate was also increased in most of the intestinal segments except the mid-distal colon. However, when the perfusates reached excessive acidification or alkalization, the rate of fluid absorption and *J*_*HCO3*_ *-* decreased in all the segments (Figure 5).

## Discussion

To understand the physiology and pathophysiology of intestinal fluid transport and bicarbonate secretion, many animal models *in vivo* have been made (Mourad, 2004; Xia et al., 2014; Jung et al., 2014; Tan et al., 2021). Among them, intestinal loop and steady-state intestinal perfusion systems can represent the proper tools. The intestinal loop technique can be set up easily but with a drawback that only one data from every segment of individual mouse could be acquired in every experiment. However, the steady-state intestinal perfusion technique allows these measurements in the different intestinal segments of interest, repeatedly (Xia et al., 2014; McHugh et al., 2018). In our study, the intestinal segments were perfused three times in every experiment, and the values were averaged to get the result, eventually. Nevertheless, many studies reported intestinal fluid absorption and bicarbonate secretion by using this system, but detailed procedures as well as many key indicators during the experimental process were not spotted (Singh et al., 2009).

In the present study, we gave the details of experimental procedures as well as key signs, such as blood gas, hematocrit, blood pressure, and heart rate, and used this system to measure intestinal fluid transport and bicarbonate secretion. In this system, the anesthetized, tracheal intubated and artificially ventilated mice could be maintained for more than 3 h. Our study showed that the status of mice was unstable with low MAP, high heart rate and hematocrit, and mild systemic acidosis after the surgery was completed. This could be explained by fluid loss during the preparation for intestinal segments because the abdomen was opened, which could cause the insufficiency of effective blood volume. In addition, fluid loss from the respiratory tract could also result in a low blood volume (Kang et al., 2016). Moreover, effects of the anesthetic drug on the cardiovascular system are also in consideration (Choi et al., 2022). However, these indicators improved to the normal level after 30 min by virtue of fluid supplementation from the carotid artery and the femoral vein and were kept stable for approximately 2 h, which can enable us to have enough time to observe the fluid movement and bicarbonate secretion. However, mice status got worse at the 180 min time point with signs of blood pressure decreasing and systemic acidosis. Not surprisingly, a long time of mechanical ventilation and anesthesia would break homeostasis of the internal environment inevitably. Corresponding to the change of former indicators, the rate of fluid absorption and bicarbonate secretion was low at the beginning of surgery completion and increased to the plateau after 30 min, and the rate of them also decreased significantly at the 180 min time point, which highlighted the pivotal effect of homeostasis of the cardiovascular system on intestinal function. Taken together, our data indicated that the 30–150 min time point is an optimal time period for measurement.

Normally, to mimic the physiological condition, neutral perfusates with pH 7.4 were applied. A previous study suggested that the pH of solution could affect the fluid absorption (Tan et al., 2021). In the present study, we tested the fluid absorption and bicarbonate secretion of four kinds of perfusates with pH 6.2, 6.8, 7.4, and 8.0 in the small and large intestines. The rate of fluid absorption increased in all segments’ perfusate with pH 6.8. Regarding *J*_*HCO3*_ *-*, the rate was also increased in most of the intestinal segments expect the mid-distal colon. However, the rate of fluid absorption and *J*_*HCO3*_ *-* decreased significantly in all segments when the perfusates were either excessively acidic or alkaline. Many studies have drawn the pH profile of the intestinal tract of animals and humans (Maxwell et al., 1968; Ovesen et al., 1986; Waldman and Camilleri, 2018). This indicates that acid–base balance in the intestinal lumen is critical for normal intestinal function. Excessive acidification or alkalization of the intestinal lumen would inhibit ion transporters and channels on the intestinal epithelia resulting in the dysfunction of fluid absorption and bicarbonate secretion.

Since we figured out the optimal condition for fluid absorption and bicarbonate secretion measurement, next, we measured the rate of them in different intestinal segments. In line with the previous data, different rates of fluid absorption and bicarbonate secretion were observed in different intestinal segments. Many studies have shown the different expression patterns of ion transporters and channels on different segments of intestinal epithelia (Kato and Romero, 2011; Seidler and Nikolovska, 2019), which could robustly explain the different rate of fluid transport and acid–base secretion in different locations of the jejunum, ileum, and colon.

Undoubtedly, this animal model can provide substantial information. However, it requires a high level of technical expertise. Like the tracheal intubation of mice, the insertion of small catheters into the carotid artery and the femoral vein and the meticulous proceeding during intestinal surgery to avoid the massive loss of blood are challenging. Optimal control of blood pressure and breathing rate is also needed. Furthermore, the effects of the anesthetic drug on the intestinal function cannot be ruled out because all animals were anesthetized in our *in vivo* experiments.

In summary, in the present study, we gave the detailed methods of the steady-state intestinal perfusion system in measuring fluid absorption and bicarbonate secretion. In addition, the key indicators including the blood pressure, hematocrit, blood gas, and heart rate were also presented. Moreover, a series of stable data of fluid absorption and bicarbonate secretion both in small and large intestines strongly supported the reliability of this system. Although some disadvantages are inevitable, it is currently a very, if not the most, valuable method to help us understand the intestinal fluid transport *in vivo*.

## Data Availability

The original contributions presented in the study are included in the article/Supplementary Material; further inquiries can be directed to the corresponding author.
